# The Effects of Mindfulness-Based Mandala Coloring, Made in Nature, on Chronic Widespread Musculoskeletal Pain: Randomized Trial

**DOI:** 10.3390/healthcare9060642

**Published:** 2021-05-28

**Authors:** Han Choi, Suk-Chan Hahm, Yo-Han Jeon, Jin-Woo Han, Soo-Yeon Kim, Jong-Min Woo

**Affiliations:** 1 Graduate School of Art Therapy, Cha University, Seongnam 13488, Korea; arttherapyhan@cha.ac.kr; 2 Graduate School of Integrative Medicine, Cha University, Seongnam 13488, Korea; schahm@cha.ac.kr; 3 Department of Child Psychology and Education, Sungkyunkwan University, Seoul 03063, Korea; creativitylex@gmail.com; 4 Faculty of Public Health, Wonkwang University Graduate School, Iksan 54538, Korea; eurochestnuter@gmail.com; 5 Department of Medicine, Graduate School, Cha University, Seongnam 13488, Korea; sarang2x2@naver.com; 6 Seoul Mental Health Clinic, Seoul 06149, Korea

**Keywords:** pain, mindfulness-based intervention, art therapy, nature, stress, mandala

## Abstract

This study aimed to investigate the effects of mindfulness-based mandala coloring made within nature on individuals with chronic widespread musculoskeletal pain (CWP). Thirty-six participants were randomly allocated. In the experimental group, identical interventions and procedures were administered for each experiment. The control group members were untreated and remained in an urban environment. Overall, the experiment showed significant improvements in tender points (*f* = 8.791, *p* = 0.006), total stress level (*f* = 14.570, *p* = 0.001), depressive symptoms (*f* = 15.205, *p* = 0.001), anger symptoms (*f* = 7.263, *p* = 0.011) and salivary cortisol (*f* = 10.619, *p* = 0.003) in the experimental group. The results reflect that MBMC within nature is effective in reducing pain, psychological stress responses, and cortisol levels in individuals with CWP. The positive results could be a product of the experimental design rather than the treatment itself. A rigorous experimental design provides better understanding of MBMC within nature.

## 1. Introduction

Chronic widespread musculoskeletal pain (CWP) is defined as continuous pain felt in at least four parts of the body, including above and below the waist, left and right of the central axis, and near the spine, and lasting for over three months along with emotional distress [1]. In the general population, the prevalence of CWP ranges from 10 to 25% [2,3]. CWP has been reported to be accompanied by psychological complications, including stress [4], anxiety, depression [5], and fatigue [6,7].

Studies have demonstrated the effectiveness of various non-pharmacological pain management interventions for conditions including CWP [8,9,10]. Studies have shown that mindfulness-based interventions are effective in reducing pain, anxiety, depression, and stress [11,12,13,14]. Mindfulness-based stress reduction (MBSR) [15] is one of such interventions first introduced to treat chronic pain [16]. Subsequently, MBSR was expanded to mindfulness-based art therapy (MBAT), which combines the elements of MBSR [17] and art therapy. Art therapy is a form of psychotherapy that uses imagery and visual expression as a means of communication. Therapeutically, art facilitates self-realization and rediscovering a more evident self [18]. There are a few art therapy studies that reported chronic pain effects for various population groups and medical conditions [19,20,21,22,23]. The therapeutic factors revealed in art therapy studies for pain management are as follows. First, art making evolved to provide the individual with a way to increase the security of relational attachment, when their pain experiences are shared with the art therapist [19,21,22]. Second, group art therapy is useful for emotional and psychological support when their pain experiences are shared with others [23]. MBAT aims to connect the creative process of art making with the awareness of inner self and emotions [24]. MBAT reportedly reduces distress symptoms [24] while improving the psychological stability [17,25] and quality of life [26] of patients with oncological conditions [27].

A mandala, meaning “circles” in Sanskrit, is a ritual and a spiritual symbol. The complexity and circular and geometric patterns of a mandala are therapeutically beneficial, promoting the state of mindfulness [28]. There are conflicting results of pre-line coloring activity versus free-choice coloring activity in art therapy studies [29]. Selective studies have indicated that the pre-line coloring activity shows a range of beneficial effects from reducing stress [30,31] to stabilizing mood [32] when art therapy is facilitated by the art therapist. Therapeutically, art is a natural activity familiar to children [33]. In adults, being overwhelmed with the blank paper and the fear of being judged [34] are obstacle factors.

The positive benefits of exposure to nature have been reported for various health conditions. Studies have shown that contact with nature is effective for conditions related to stress [35,36], fatigue [37] and depression [38]. Among studies that have investigated pain management and alleviation, those on the psychological and physiological benefits of natural environments and the broader context of nature have reported no adverse effects [39]. Similarly, short-term exposure to natural environments has been shown to reduce levels of inflammatory biomarkers, including interleukin-6 and tumor necrosis factor-alpha [40,41]. Exposure to nature alone [42] and a combination of art activities and exposure to nature have reportedly enhanced creativity [43]. A pioneering study reported that the viewing of nature by patients while they were in postsurgical hospital care reduced the patients’ pain and enhanced their recoveries [44]. In addition, patients subjected to a looking at images depicting natural elements while in hospital wards during bronchoscopies reported reduced pain [45]. Furthermore, it was found that incorporating nature into art therapy leads to an increase in emotional and psychological well-being [46,47].

The impact of mindfulness-based mandala coloring (MBMC) made within nature on CWP management has not been reported yet, to the best of our knowledge. Thus, the primary objective of this study is to investigate the prospects of MBMC made within nature as a mind–body and management strategy in participants with CWP, by specifically investigating the number of tender points compared to urban stimuli. The secondary objective is to investigate fatigue severity, stress, and cortisol levels compared to urban stimuli.

## 2. Materials and Methods

### 2.1. Sample Size Calculation

A sample size calculation was carried out prior to conducting this research. The sample size calculation, based on the ANOVA result, effect size (*f* = 0.070), and a power of 80%, was conducted by using G*Power version 3.1.9.7 (Universität Düsseldorf, Düsseldorf, Germany) [48]. According to a previous study on nature-based therapy on CWP [39], the estimated sample size was calculated to be 42 participants.

### 2.2. Participants

The participants were selected from a sampling frame of building and facilities management employees with CWP in the Central Business District of Seoul, Republic of Korea. Participants were selected because musculoskeletal pain conditions were prevalent and associated with work demands, such as awkward postures and repetitive movements among maintenance workers [49].

The inclusion criteria for this study were (a) adults aged 19 to 64 years having experienced pain for three months or more in at least four tender points according to the Manual Tender Point Survey (MTPS) [50], and (b) no pre-existing conditions such as substance abuse and psychiatric disorders. The exclusion criteria were (a) pre-existing pain conditions other than CWP, (b) history of outdoor allergens (e.g., to dust, pollen, trees), in which existing allergic disease can exacerbate discomfort and psychological stress [51], (c) ongoing arts or mindfulness-based therapies during the experimental period, and (d) requiring pain-related medication on a daily basis. Participants were recruited via self-referral. Print advertisements were posted on the workplace noticeboard. Participants made a screening appointment by telephone. By using a screening test prior to enrollment, a rheumatologist recruited participants and examined their reported numbers of tender points based on MTPS, pre-existing pain conditions, and history of outdoor allergens. Then, a psychiatrist assessed pre-existing psychiatric disorders. Finally, participants’ previous creative experiences were collected. For the first experiment, nine participants received MBMC made within nature, while six did not. For the second experiment, twelve and nine participants, respectively, were allocated to the experimental and control groups.

### 2.3. Instruments and Data Collection

#### 2.3.1. Primary Instruments

Tender points were assessed with the MTPS [50], an illustration of the 18 places on the body that are sensitive to pressure as specified by the American College of Rheumatology [52]. Permission to use the MTPS was obtained from the developer.

#### 2.3.2. Secondary Instruments

The Fatigue Severity Scale (FSS) [53], a 9-item self-report questionnaire of symptoms measured on a 7-point Likert scale, was used to measure the participants’ fatigue symptoms. Total scores of less than 36 suggested that the participants were not experiencing fatigue, while scores of 36 or more suggested that they were [53]. The FSS was validated in the Republic of Korea with excellent reliability (Cronbach’s α = 0.93) [54].

The Stress Response Inventory—Modified Form (SRI-MF) [55] is a 22-item self-report questionnaire of stress symptoms measured on a 5-point Likert scale. It includes subscales of depressive, somatization, and anger symptoms (Cronbach’s *α* = 0.94) [55].

Levels of the stress hormone cortisol, a non-invasive biomarker, were measured in saliva, which was collected with Salivette^®^ tubes (Sarstedt AG & Co., Nümbrecht, Germany). The tubes were refrigerated at −20 °C until analysis. The saliva samples were sent to Meditree Co., Ltd. (Seoul, Korea) for analysis according to the manufacturer’s protocols. Pre- and post-test saliva samples were collected in the morning as baseline because salivary cortisol levels are highly sensitive to time of day, especially in the morning [56]. Baseline cortisol levels should be taken immediately upon waking as cortisol spikes within the early hours of the morning or rising [57]. A registered clinical pathologist collected saliva samples.

A portable multifunctional measuring instrument (MI 6401 Poly, METREL D.D., Horjul, Slovenia) was used at one-min intervals to evaluate the climate environment (temperature, humidity, and wind speed). To calculate the duration of sunlight, climate information was used from the Korea Meteorological Administration (KMA) (https://web.kma.go.kr/eng/index.jsp accessed on 6 May 2016). To measure levels of airborne natural volatile organic compounds (NVOCs), a portable air absorption instrument (MP-Sigma 300N, Sibata Scientific Technology, Ltd., Soka, Japan) was used with a Tenax TA tube (Sigma-Aldrich Co. LLC, St. Louis, MO, USA). Over two hours at a stream velocity of 100 mL/min, 9-L samples were collected. Two samples were collected at dawn, then at midday, and at dusk. The samples were analyzed using gas chromatography–mass spectrometry at the National Instrumentation Center for Environmental Management of Seoul National University.

### 2.4. Procedure

Thirty-nine participants were randomly assigned to the experimental group or control group using the stratified randomization method in order to control selection bias. Moreover, to reduce detection bias, this study used the blinding of outcome assessors. MBMC made within nature was administered to the experimental group in September, 2016 (first experiment), and to the second group in September, 2017 (second experiment). Due to a deficient small pilot sample size in the first experiment, a second experiment was conducted. In an attempt to control or minimize extraneous variables such as weather conditions, the second was conducted exactly one year apart. Experiment dates were determined by climate data, including historical weather data provided by the KMA for the two experimental sites. Identical instruments and procedures were followed for pre- and post-testing. Baseline saliva cortisol samples were collected, and psychological instruments were gathered in case report forms during pre-testing in an urban environment prior to participants’ visits to the experimental site. Post-testing saliva samples were collected in the morning between 07:30 and 08:00 a day after intervention.

The participants were not allowed to consume food or caffeinated beverages two hours before the experiments because caffeine and food intake are known to affect the measurement of saliva cortisol [58,59]. Participants in the experiments assembled and then departed from the hospital to the experimental site via charter bus. Estimated travel time took an hour one way trip journey and two hours round trip journey.

Identical therapeutic activities were administered for both experimental groups at the experiment site. Both experimental groups were given identical diets (meals, snacks, and beverages). When they were actively involved in MBMC made within nature, levels of NVOCs and meteorological components were measured. After the intervention ended, post-therapy instruments identical to the pre-test were used. Comparatively, the control group remained outdoors in the urban environment. Both control and experimental group participants were provided with identical diets. Pre- and post- instruments and procedures were identical for both groups. The control group visited Seoul Paik Hospital for the pre- and post-test measurements, which were identical to the measurements conducted in both experimental groups. A non-guided sightseeing bus tour for four hours in an urban environment was administered as urban stimulation for the control group. While engaged in the non-guided sightseeing bus tour, participants were controlled for excessive movement and use of mobile devices and paper-based media. The time schedule of the experimental procedure is presented in Table 1.

### 2.5. Interventions

The experimental site for MBMC made within nature was in the Natural Recreation Forest in Yangpyeong County of Gyeonggi Province, about 80 km from Seoul. During a day, MBMC made within nature was aimed at providing mind and body awareness, relaxation, and stress relief to participants with CWP. There was no previous study of the combination of mindfulness-based and art therapy in nature. Therefore, mindfulness-based mandala coloring made within nature was conducted within the framework of mindfulness-based art activities in terms of coloring materials provided to participants [20]. The group therapy format was rationalized based on the evidence-provided guidance that group support is beneficial [23]. MBMC within nature consisting of a mandala drawing lasted for four hours. A pre-drawn mandala was chosen for this study because the majority of participants had limited experience in art making, and such drawings can provide a sense of security when using art materials [60].

**Figure 1:**
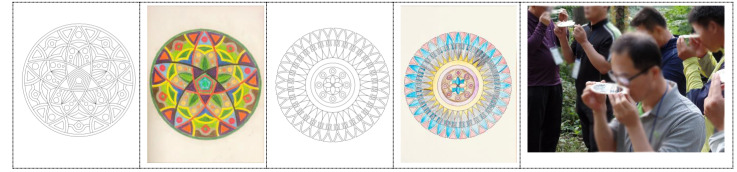
Samples of pre-line mandala, completed mandala drawings and sky mirror activity.

The procedure of MBMC made within nature included (a) raising awareness of the natural surroundings, (b) closely observing the natural surroundings through a portable sky mirror, (c) selecting an “8.5 × 11” pre-line paper mandala template, (d) coloring the mandala with colored pencils while monitoring changes in emotional state, (e) observing the natural surroundings, and (f) sharing the experience with others. Figure 1 demonstrates intervention procedure. Total MBMC session duration was four hours over a day because mandala coloring can be used for short-term treatment [25]. In the experimental group, mindfulness-based mandala coloring was conducted by a registered art psychotherapist.

### 2.6. Ethical Considerations

This experiment was approved by the Institutional Review Board of Seoul Paik Hospital of Inje University. The purposes and procedures of the studies were thoroughly explained to the participants, who were free to withdraw from the study at any time. All participants provided written informed consent prior to the study.

### 2.7. Data Analyses

All statistical analyses were performed using SPSS 21.0 (IBM Corporation, Armonk, NY, USA). The normality of collected data was verified using a Shapiro–Wilk test, and the dependent variables showed a normal distribution (*p* > 0.05). The continuous variables were analyzed with two-way repeated measures ANOVA and paired-sample *t*-tests. Nominal and environmental variables that were not normally distributed were analyzed with a Cochran–Mantel–Haenszel test and Mann–Whitney U-tests.

## 3. Results

### 3.1. Participant Characteristics

**Figure 2:**
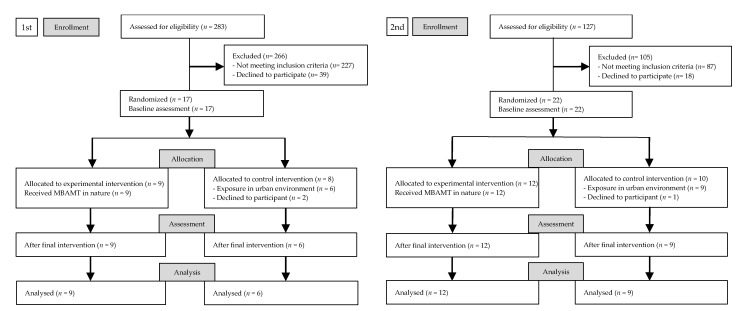
CONSORT flow diagram of first and second experiment.

A total of 36 patients completed the study. Figure 2 demonstrates the CONSORT flow diagram of participants through the trial. Participants (*n* = 410) completed a screening that comprised questions addressing numbers of tender points and their willingness to participate in MBMC made within nature. For the first experiment, 283 participants were surveyed from August to September 2016. For the second experiment, 127 participants were surveyed from July to August 2017. Of the 410 participants, 43 fulfilled the inclusion criteria and were identified as eligible candidates and 371 did not fulfill the inclusion criteria. Of these, 39 agreed to participate in the studies (first experiment, *n* = 17; second experiment, *n* = 22). After randomization, three patients declined to participate (first experiment, *n* = 2; second experiment, *n* = 1).

An overview of the demographic characteristics of the participants is presented in Table 2. The experimental and control groups were compared, and there were no significant differences in any of their demographic characteristics. Participants’ employment status was not queried.

### 3.2. Environmental and Meteorological Results

Table 3 shows that the results of the two experiments were not significantly different (*p* > 0.05), indicating that the confounding variables of the environment were controlled.

### 3.3. Physiological and Psychological Variables in the Overall Experiment

A two-way repeated measures ANOVA was conducted to determine the effectiveness of MBMC in the first and second experiments combined. The numbers of tender points (*f* = 8.791, *p* = 0.006) and salivary cortisol (*f* = 10.619, *p* = 0.003) have statistically significant interaction effects. In addition, total SRI-MF scores (*f* = 14.570, *p* = 0.001), somatization symptoms (*f* = 6.374, *p* = 0.016), depressive symptoms (*f* = 15.205, *p* = 0.000), and anger symptoms (*f* = 7.263, *p* = 0.011) were significant. However, the FSS (*f* = 2.370, *p* = 0.1333) was not significantly different. Examination of the between-group effect sizes indicated large effect sizes for tender points (partial eta squared = 0.205), somatization symptoms of SRI-MF (partial eta squared = 0.158), depressive symptoms of SRI-MF (partial eta squared = 0.309), anger symptoms of SRI-MF (partial eta squared = 0.176), total SRI-MF score (partial eta squared = 0.300), and salivary cortisol (partial eta squared = 0.238). The effect sizes of FSS (partial eta squared = 0.065) were moderate. As shown in Table 4, the paired *t*-tests revealed significant changes in all variables except FFS in the experimental group.

## 4. Discussion

This study showed that MBMC within nature relieved pain and psychological complications notably in individuals with CWP as compared to participants who remained in an urban environment and did not receive MBMC. Although this study presents promising results in terms of statistical powers, careful interpretations are required to evaluate improvements. In terms of factors affecting experimental design, this study has a limited sample size and is open-label, which raises potential selection bias that favors the treatment. There were two separate tests conducted over two years, and the designed control condition that relied on a subjective primary outcome may have influenced improvements rather than the treatment itself. The proposed treatment method integrated with a creative approach and contact with nature is relatively novel [46]. Therefore, as pain management and stress relief in patients with CWP, MBMC within nature should be tested as an alternative and complementary therapeutic intervention for the management of patients with CWP.

The percentage of identified eligible candidates was 9.5% (*n* = 39). Conversely, the percentage of non-eligible candidates was 90.5% (*n* = 314). The exact reason for the high percentage of screening test rates is unknown. Of those who did not satisfy the inclusion criteria, 68.7% (*n* = 216) were found not to have CWP. Their pain condition was continuous, and there were less than three tender points according to the MTPS assessment. A high interest in participation in MBMC or nature therapy or MBMC within nature, an opportunity for medical evaluation and consultation with doctors, and looking for a free retreat away from the city might be possible explanations for the high percentage of screening test rates.

In the first experiment, cortisol levels, total stress response score, and somatization, anger, and depressive symptoms significantly improved. Similarly, in the second experiment, tender points, total stress response scores, depressive and anger symptoms, and cortisol levels improved considerably. This finding is congruent with a study that reported that individuals receiving mindfulness-based art therapy reported significant decreases in pain and negative emotions [17,27]. Furthermore, in this study, significant improvements were observed in terms of decreased stress through contact with nature, which is consistent with the findings of previous studies [35,36]. The outcomes of the present study bring the possibility of expanding the evidence in creative and nature-integrated treatment methods [46] and add to the existing knowledge [39] that has verified the effects of nature-based therapy on CWP with a focus on pain intensity, immune competence, and quality of life. A significant improvement in the number of tender points with MBMC within nature was observed in the first experiment. In the second experiment, a considerable improvement in pain was not observed, but the number of tender points decreased in pre-post values in between groups. Thus, the results showed that MBMC within nature relieves CWP. However, MBMC within nature failed to improve fatigue in the experimental group as compared to the control group. Considering that travel to the experimental sites could increase fatigue, it is possible that the effects of MBMC within nature on relaxation and reducing fatigue might have been counterbalanced. The decline in salivary cortisol as an objective measurement within the experimental group was significantly effective. The result suggested that the MBMC participants became less stressed physiologically. This result was in compliance with results regarding psychological stress. Hence, MBMC seemed to aid participants in stress relief.

Overall, MBMC within nature could not verify therapeutic factors identified from art therapy studies on pain management [19,20,21,22,23]. In terms of art therapy providing security of relational attachment for individuals with chronic pain [19,21,22], MBMC within nature would take this into account because this study was implemented on a short-term basis. In terms of art therapy useful for emotional and psychological support for individuals with chronic pain [23], MBMC within nature may have influenced the results because it was implemented on a group basis and the therapeutic protocol includes sharing the experience with others.

Extending to non-pharmacological pain management treatment approaches regarding CWP, those studies differ from this study in terms of study design, treatment strategies, the length and frequency of treatment, and ethnicity. Therefore, the results of this study should be interpreted with caution. In indirect comparisons, the results were consistent with a psychological treatment [10], in which short-term treatment is possibly beneficial. Yet again, the results were consistent with multidisciplinary treatment approaches [8,9], suggesting effectiveness in pain management. Pre-treatment preferences should be accounted for in participants’ subjective treatment in order to increase treatment adherence for multidisciplinary treatment approaches [9].

This study consisted of two separate experiments. The results were significant due to the reproducibility of the experiment. Extraneous factors, including weather conditions, temperature, humidity, sunshine duration, and environmental elements that could have affected MBMC, were similar. This study could not isolate specific effects of the nature and mindfulness-based art therapy components of the intervention.

This study has some limitations. First, it was performed with a small sample size and the intervention was applied for a short period. A larger sample with an appropriate sample size, calculation sizes, and study design with longer-term application of the intervention may provide more substantial evidence for the effectiveness of MBMC within nature for CWP patients. Second, this study lacks generalizability in terms of ethnicity and race. A random sample from the target population was collected, which may have led to the generalization of results. All participants belonged to East Asian ethnic groups native to Korea. Therefore, a confirmation study is required to verify the general effects of MBMC within nature on CWP in wider ethnic and race groups. Third, a combined and multidisciplinary approach, rather than a single therapeutic activity, was performed in this study. Thus, the results cannot verify the effects of each therapeutic activity. Fourth, in terms of study design, this study used a passive control group as the only comparison. An active control design comparing nature-based therapy or art therapy may provide better evidence on the effects of MBMC within nature for CWP. Fifth, objective outcome measures such as the heart rate variability would evaluate real-time changes in physiological conditions via MBMC within nature since this study has relied on subjective primary outcomes. Finally, there was no follow-up, which would be important, especially in this short-time therapy conducted on the completion of MBMC within nature for CWP. Therefore, a follow-up study to assess stress conditions at home or work would provide better insight into the effects of MBMC within nature on CWP.

## 5. Conclusions

This study examined the effects of MBMC within nature. The results indicated that mindfulness-based art therapy and contact with nature were statically favorable in reducing chronic pain and stress levels in individuals suffering from CWP. It is suggested that MBMC could be introduced in specific conditions, mild weather conditions and natural environments, and when supervised by an art therapist, it could serve as a possible therapeutic choice for musculoskeletal or chronic pain conditions in patients. A rigorous experimental design would provide a better understanding of MBMC within nature.

## Figures and Tables

**Table 1:** Time schedule of experimental procedure.

Time Schedule	Experimental Group		Control Group	
	Activity	Location	Activity	Location
07:30–08:00	Pre-test	Hospital	Pre-test	Hospital
09:30–13:30	Mindfulness-based Mandala Coloring	Natural Recreation Forest	Sightseeing Tour	Urban Area
14:00–14:30	Post-test	Hospital	Post-test	Hospital

**Table 2:** Demographic characteristics of the experimental and control groups.

Demographic Characteristics	First Experiment				Second Experiment				*p*
	Experimental Group		Control Group		Experimental Group		Control Group		
	M (SD)	*n* (%)	M (SD)	*n* (%)	M (SD)	*n* (%)	M (SD)	*n* (%)	
Ethnicity									
Korean	9 (100%) ^a^		6 (100%) ^a^		12 (100%) ^a^		9 (100%) ^a^		
Gender									
Male	5 (55.6%) ^a^		4 (66.7%) ^a^		9 (75.0%) ^a^		5 (55.6%) ^a^		0.676
Female	4 (44.4%) ^a^		2 (33.3%) ^a^		3 (25.0%) ^a^		4 (44.4%) ^a^		
Age (years)	41.67 (4.64) ^b^		39.17 (6.37) ^b^		47.42 (5.52) ^b^		43.56 (4.93) ^b^		0.712
Height (cm)	166.56 (8.90) ^b^		170.17 (8.40) ^b^		168.67 (7.58) ^b^		166.67 (7.18) ^b^		0.313
Weight (kg)	67.89 (12.03) ^b^		66.83 (10.53) ^b^		71.67 (16.23) ^b^		68.11 (13.67) ^b^		0.794
Previous experience on treatment									
Yes	6 (66.7%) ^a^		4 (66.7%) ^a^		6 (50.0%) ^a^		7 (77.8%) ^a^		0.748
No	3 (33.3%) ^a^		2 (33.3%) ^a^		6 (50.0%) ^a^		2 (22.2%) ^a^		

Notes. ^a^ Data presented as number (*n*), percentage (%). ^b^ Data presented as mean (M) ± standard deviation (SD) cm = centimeter. kg = kilogram.

**Table 3:** Environmental and meteorological variables in the first and the second experiment.

Variables	First Experiment	Second Experiment	*Z*	*p*
	M (SD)	M (SD)		
Temperature (°C)	19.33 (1.91)	21.18 (1.19)	−1.443	0.200
Diurnal Temperature Range (h)	11.88 (2.95)	12.83 (2.06)	−0.289	0.886
Relative humidity (%)	66.55 (11.64)	60.48 (21.62)	−0.289	0.886
Sunshine duration (h)	7.75 (3.08)	9.63 (1.81)	−0.866	0.486
Average wind speed (m/s)	1.28 (0.55)	1.53 (0.80)	−0.441	0.686
NVOCs (mg/m^3^)	1.00 (1.04)	1.65 (0.83)	−1.680	0.093
Dust (mg/m^3^)	2.44 (0.02)	2.43 (0.01)	−0.420	0.672

Notes. Data presented as mean (M) ± standard deviation (SD). NVOCs = natural volatile organic compounds. °C = Celsius. h = Hour. % = Percentage. m/s = meter per second. mg/m^3^ = microgram/cubic meter.

**Table 4:** Comparison of instruments between and within experimental and control groups in the overall experiment.

Variables	Pre-Test	Post-Test	*f*	*p*	Post Hoc		
	M ^a^ (SD ^b^)	M ^a^ (SD ^b^)			*t*	*Df*	*p*
Tender Point							
Experimental	7.52 (3.20)	3.81 (2.42)	8.791	0.006	7.526 ***	20	0.001
Control	6.73 (2.34)	5.67 (2.23)			1.331	14	0.205
FSS							
Experimental	41.19 (9.13)	37.90 (11.73)	2.370	0.133	1.381	20	0.183
Control	42.93 (11.36)	44.73 (13.82)			−0.885	14	0.391
SRI-MF							
Somatization Symptom							
Experimental	11.71 (8.31)	7.29 (8.93)	6.374	0.016	4.217 ***	20	0.001
Control	13.53 (10.51)	12.87 (11.07)			0.688	14	0.503
Depressive Symptom							
Experimental	9.48 (8.73)	6.05 (8.82)	15.205	0.000	5.056 ***	20	0.001
Control	9.27 (10.14)	9.73 (10.38)			−0.664	14	0.517
Anger Symptom							
Experimental	5.57 (4.63)	3.67 (5.16)	7.263	0.011	4.119 **	20	0.001
Control	7.80 (6.20)	7.47 (6.45)			1.435	14	0.173
Total Stress Level							
Experimental	28.10 (21.80)	18.05 (23.47)	14.570	0.001	5.691 ***	20	0.001
Control	32.13 (26.63)	31.53 (27.74)			0.384	14	0.707
Cortisol							
Experimental	0.24 (0.15)	0.18 (0.13)	10.619	0.003	4.045 **	20	0.001
Control	0.27 (0.20)	0.30 (0.21)			−1.206	14	0.248

Notes. ^a^ Mean, ^b^ Standard deviation, ** Significant at *p* < 0.01, *** Significant at *p* < 0.001, FSS = fatigue severity scale. SRI-MF = Stress Response Inventory—Modified Form.

## Data Availability

The data that support the findings of this study are available from the corresponding author upon reasonable request.
